# Novel reversible and switchable electrolytes based on magneto-rheology

**DOI:** 10.1038/srep15663

**Published:** 2015-10-23

**Authors:** Jie Ding, Gangrou Peng, Kewei Shu, Caiyun Wang, Tongfei Tian, Wenrong Yang, Yuanchao Zhang, Gordon G. Wallace, Weihua Li

**Affiliations:** 1 Land Division, Defence Science and Technology Group, 506 Lorimer Street, Fishermans Bend, VIC 3207, Australia; 2 School of Mechanical, Material and Mechatronic Engineering, University of Wollongong, Wollongong, NSW 2522, Australia; 3 ARC Centre of Excellence for Electromaterials Science, Intelligent Polymer Research Institute, University of Wollongong, Wollongong, NSW 2522, Australia; 4 School of Life and Environmental Sciences, Deakin University, Geelong, VIC 3217, Australia

## Abstract

Replacing organic liquid electrolytes with solid electrolytes has led to a new perspective on batteries, enabling high-energy battery chemistry with intrinsically safe cell designs. However, most solid/gel electrolytes are easily deformed; under extreme deformation, leakage and/or short-circuiting can occur. Here, we report a novel magneto-rheological electrolyte (MR electrolyte) that responds to changes in an external magnetic field; the electrolyte exhibits low viscosity in the absence of a magnetic field and increased viscosity or a solid-like phase in the presence of a magnetic field. This change from a liquid to solid does not significantly change the conductivity of the MR electrolyte. This work introduces a new class of magnetically sensitive solid electrolytes that can enhance impact resistance and prevent leakage from electronic devices through reversible active switching of their mechanical properties.

Energy storage devices require a reliable electrolyte medium^1^. Organic liquid electrolytes provide superior power densities and have been widely used in batteries and supercapacitors^2^,^3^,^4^. However, the use of organic liquid electrolytes poses safety issues because they are highly flammable and unstable when exposed to air^5^,^6^. Consequently, solid-state electrolytes have been studied^7^,^8^,^9^. The main advantages of solid electrolytes over liquid electrolytes are their environmental stability and improved impact resistance^10^,^11^; however, the improvement in mechanical strength is offset by a significant reduction in conductivity; a shortcoming that hinders the use of solid-state electrolyte technology.

Magneto-rheology refers to the reversible and rapid change of magneto-rheological fluids (MRFs) from liquid to semi-solid or solid when an external magnetic field is applied^12^. The phase change is attributed to the dipolar interactions introduced by the external magnetic force, which initiates the formation of a chain-like alignment^13^. MRFs are an important class of smart materials that are used in a variety of applications, such as vibration control, torque transmission^14^,^15^,^16^, robotics and micro-electronic devices^17^,^18^. MRFs contain soft magnetic particles in a fluid, such as mineral oils, silicone oils, polyesters, polyether, synthetic hydrocarbons or water. The nature of the solvents and any added surfactants contribute to the stabilities^12^ and durabilities^19^ of the dispersions. Ionic liquids (ILs) have also been used as an effective carrier fluid for MRFs for durability^19^ and improving stability^20^. ILs are salts that are fluid at ambient temperatures^21^,^22^ and are widely used in energy storage devices^23^,^24^,^25^ due to their negligible vapour pressures, thermal stabilities and non-flammability^26^,^27^,^28^.

In this work, we report a novel reversible solid electrolyte based on magneto-rheology, which combined excellent conductivity and mechanical properties that are responsive to field changes. This novel magneto-rheological electrolyte (MR electrolyte) was generated by dispersing magnetic nanoparticles in an IL, 1-ethyl-3-methylimidazolium bis(trifluoromethylsulfonyl)imide (EMITFSI). Fumed silica nanoparticles were used to stabilise the magnetic particles. The proposed MR electrolyte exhibited field-dependent properties and excellent conductivity. The viscosity of the MR electrolyte could be controlled by varying the magnetic field intensity. The effect of the magnetic field on the conductivity of the MR electrolyte was investigated. The conductivity remained virtually unchanged during state changes, even when the electrolyte formed a viscoelastic solid. The performance of supercapacitors using such an MR electrolyte was also studied under the influence of an external magnetic field.

## Results

### Core/shell magnetic nanoparticles

Magnetic properties and an absence of redox activity are essential requirements for the nanoparticles used to generate MR electrolytes. The dispersed magnetic particles applied in this research were magnetic nanoparticles (Fe_3_O_4_) coated with a layer of silica. This layer improved dispersion stability^20^ when the particles were added to the IL. The silica layer also insulated the redox activity of the iron-containing nanoparticles.

**Figure 1:**
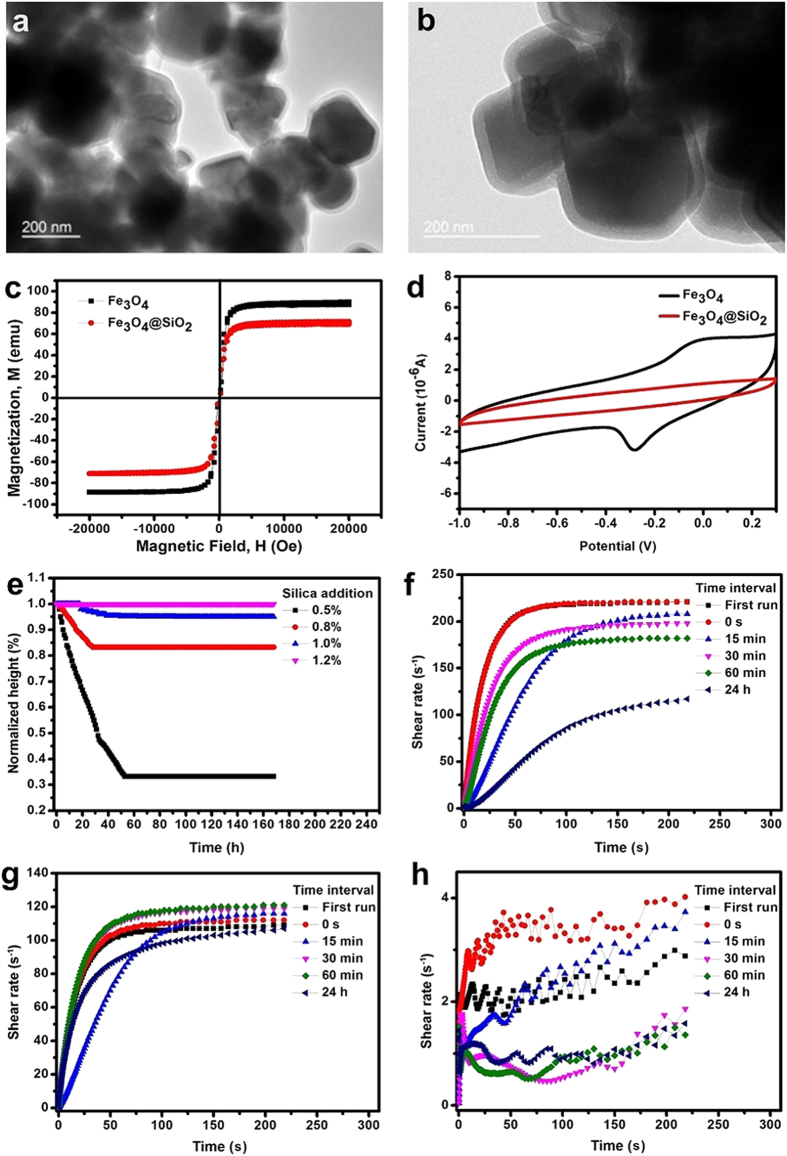
Properties of the core/shell magnetic nanoparticles and MR electrolyte samples. (**a**,**b**) TEM images of particles and clusters. (**c**) Vibrating sample magnetometer (VSM) results of magnetic nanoparticles with and without silica coatings. (**d**) Cyclic voltammetry (CV) of a carbon electrode in a 0.1 M phosphate buffer solution with Fe_3_O_4_ or silica-coated Fe_3_O_4_ nanoparticles at a scan rate of 50 mV/s over the range from −1.0 V to 0.3 V. (**e**) Normalised heights of the MR electrolytes vs. time. (**f**–**h**) Redispersion results for the MR electrolytes with (**f**) 0.8 wt% (the curves of the first 2 test runs were overlapping), (**g**) 1.0 wt% and (**h**) 1.2 wt% magnetic nanoparticles.

As revealed by TEM images (Fig. 1a,b), the cubic magnetic nanoparticles were completely covered by a transparent layer of silica with a thickness of approximately 12–15 nm. The cores of the magnetic cubic particles were in the range of 50–200 nm. The results of the vibrating sample magnetometer (VSM) tests^29^ for the original and coated magnetic nanoparticles are shown in Fig. 1c., revealing a reduction of magnetisation due to the silica coating layer. Cyclic voltammetry carried out in aqueous electrolyte containing magnetic nanoparticles with and without a silica coating layer (Fig. 1d) showed that the coated material was no longer electroactive.

The supplementary video (Video S1) also shows that the silica-coated magnetic nanoparticles retained their magnetic properties, as indicated by the fact that they could be collected from an aqueous solution with a magnetic stick.

### Stability of the MR electrolyte

The motivation for using the silica-coated magnetic nanoparticles originated from the research of Guerrero *et al.*^30^, who proposed that the change in surface chemistry induced by the coating process could significantly increase the stability of the dispersions formed in ILs^31^. In addition, small amounts of silica nanoparticles were also used to stabilise the silica-coated magnetic nanoparticles in the MR electrolyte samples. A 5% weight fraction of silica-coated magnetic nanoparticles was used in line with other studies^20^,^32^, where it was found that the dispersions were stable for more than seven days, and the slight increase in viscosity indicated that the impact on ionic conductivity was minimal. The effect of adding bare silica nanoparticles to the electrolyte was investigated over the range of 0.5–1.2 wt%. The viscosity of the resultant samples increased with increases in the fraction of silica nanoparticles. The ability to flow was substantially suppressed with the addition of 1.2 wt% silica nanoparticles.

Two separate experiments were designed to examine the stability of the MR electrolyte: a stationary observation of sedimentation and a redispersion experiment. The result of the sedimentation observation is shown in Fig. 1e, where the normalised height, which is defined as the ratio of the solid phase height recorded during the observation period to the original height, is presented. The samples with higher fractions of silica nanoparticles were more stable. The 1.2 wt% sample exhibited almost no supernatant after the entire week-long observation period, whereas the sample with 0.5 wt% silica formed a sediment within 3–4 h, with a final normalised height of approximately 33%. The normalised heights for the samples containing 0.8 and 1.0 wt% silica nanoparticles were 85% and 95.4%, respectively. When the stable samples were inverted, they rapidly redispersed without any observable compact sedimentation at the bottom of the test tube. This result confirmed that the MR electrolyte samples exhibited outstanding colloidal stability. This behaviour is an important result because stable IL-based MRFs had previously only been prepared using ILs that possess relatively high viscosities and low conductivities^33^. The sample containing 1.2 wt% silica became highly viscous and began to show the properties of a weak gel. Stable MR electrolyte samples with 0.8 and 1.0 wt% silica nanoparticles were considered potentially more useful, as they would be expected to have greater viscosity changes after the application of an external magnetic field.

In the redispersion experiments, constant shear stress was applied for different durations to investigate the reversibility of the aggregates formed in the liquid system. If the process was reversible, the corresponding shear rates would be approximately the same; if the process was not reversible, the shear rates would be considerably smaller. Figure 1f–h show the redispersion results obtained for MR electrolyte samples with 0.8, 1.0 and 1.2 wt% silica nanoparticles, respectively. The sample with 0.5 wt% silica nanoparticles was not tested because it was unstable based on the sedimentation observations. The shear rate of the MR electrolyte with 0.8 wt% silica nanoparticles decreased by nearly 100 s^−1^ after a 24 h settling period. The shear rates were comparable to each other for the MR electrolyte with 1.0 wt% silica nanoparticles over the 24 h period. The shear rates attained for the MR electrolyte sample with 1.2 wt% silica nanoparticles were similar but considerably smaller than those observed for other samples. These results indicate that the sediments formed by the MR electrolyte sample with 0.8 wt% silica nanoparticles during test were difficult to redisperse. By contrast, the sediments formed in the MR electrolyte sample with 1.0 wt% silica nanoparticles were not compact, and simple agitation restored the electrolyte to its original state. A weak gel state was noted in the MR electrolyte with 1.2 wt% silica nanoparticles (Fig. 1h). The MR electrolyte sample with 1.0 wt% silica nanoparticles was the most appropriate liquid electrolyte possessing sound colloidal stability.

### Effect of the magnetic field

**Figure 2:**
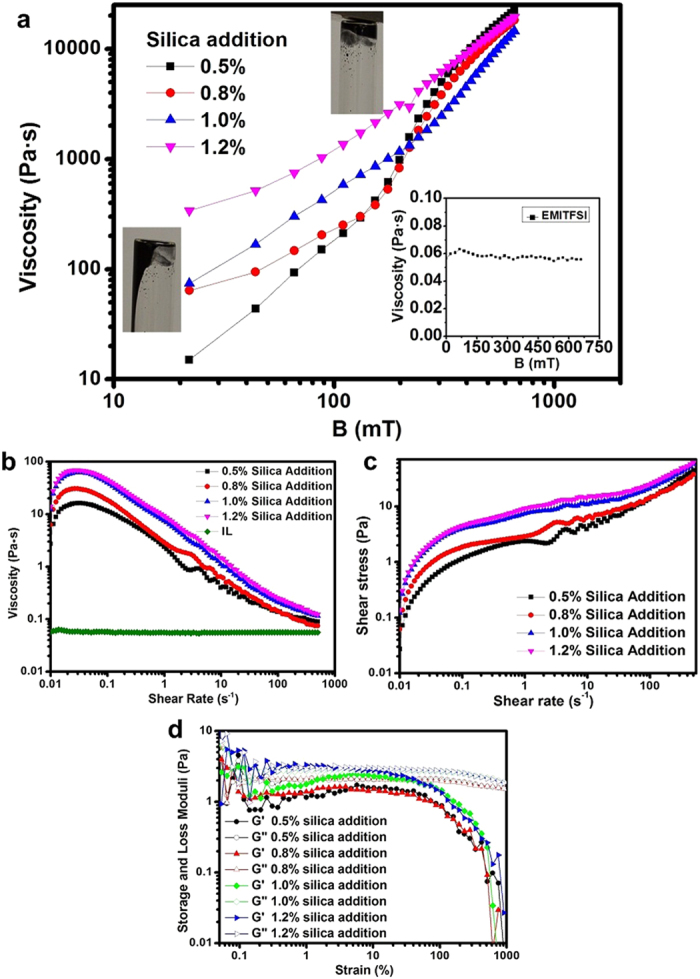
Rheological characterisation of the MR electrolytes. (**a**) Magnetic sweep tests of the MR electrolyte samples; the viscosity increased with increases in the external magnetic field, and the viscosity of EMITFSI is presented in the inset. The photograph on the left shows that the MR electrolyte is liquid at low external magnetic fields, whereas the photograph on the right indicates the solid status of the MR electrolyte at high external magnetic fields. (**b**) Shear rate sweep test results for the EMITFSI and MR electrolyte samples. (**c**) Shear stress vs. shear rate curves for the MR electrolyte samples indicating a secondary silica structure at a high shear rate. (**d**) Oscillatory dynamic experiment for MR electrolytes, where the linear viscoelastic range of the MR electrolytes can be clearly observed.

The MR electrolyte rheology was characterised via magnetic sweep, shear rate sweep and oscillatory dynamic experiments to determine whether the novel MR electrolyte was magnetically sensitive and to quantitatively establish the range of field-induced mechanical properties of the MR electrolyte. The MR electrolyte was shown to experience a dramatic increase in viscosity in Fig. 2a as the external magnetic field increased. The viscosity of EMITFSI (see inset in Fig. 2a) was shown to be constant and unaffected by external magnetic fields. The pictures in Fig. 2a show that an external magnetic field solidifies the original liquid MR electrolyte samples. This indicates that the MR electrolyte is functional over a wide range of field strengths. The ratio of the initial viscosity to the highest viscosity upon the application of an external magnetic field is a common indicator of the sensitivity to a magnetic field. The least viscous MR electrolyte sample with 0.5 wt% silica nanoparticles had a considerably higher ratio than the most viscous sample, which contained 1.2 wt% silica nanoparticles. The specific values were 1,370, 458, 329, and 102 for MR electrolyte samples with 0.5, 0.8, 1.0 and 1.2 wt% silica nanoparticles, respectively. This dramatic increase in the viscosity of the MR electrolyte during the magnetic sweep test indicates how an external magnetic field could manipulate the mechanical properties of the MR electrolyte. A video clip (Video S2) is included in the Supplementary Information to demonstrate the influence of the external magnetic field on the MR electrolyte samples.

One of the most intriguing features of IL-based colloidal dispersion systems is that the addition of silica nanoparticles could introduce unique shear-dependent non-Newtonian behaviour to the existing Newtonian carrier fluid, which is why steady shear experiments were used to investigate the shear-induced behaviour of the fabricated MR electrolyte samples. EMITFSI possessed a fairly low constant viscosity of 50 cP over the entire range of shear rates, which is an indication of typical Newtonian behaviour. However, for the MR electrolyte, shear-thinning behaviour was observed under zero-field conditions (Fig. 2b). A minor increase in viscosity was observed at a relatively high shear rate, indicative of secondary clustering due to the additional silica nanoparticles. This phenomenon was beneficial because it could be configured to serve as a fail-safe mechanism to protect the MR electrolyte against disastrous scenarios. Furthermore, the exact viscosity of the MR electrolyte could be determined under varied shear rates, whereby the addition of larger amounts of silica nanoparticles resulted in a higher initial viscosity, in agreement with direct observations. Figure 2c shows the shear stress variations in the MR electrolyte samples against shear rate, whereby the notable increase in shear stress at a relatively high shear rate agrees with the viscosity changes presented in Fig. 2b. Similar rheological behaviours occurred at higher viscosity values when an external magnetic field was applied to the MR electrolyte samples (see supplementary Figs S1 and S2).

Oscillatory dynamic experiments were performed to investigate the viscoelastic properties of the electrolyte samples; these experiments reflect the microstructures of the materials. Figure 2d shows the strain sweep dynamic test results. The storage modulus started to decrease at a strain deformation of approximately 10% for all four samples, defining an intrinsic linear viscoelastic range of the MR electrolytes, where the samples were sheared without breakage of the internal microstructure. This range was substantially wider than for typical MRFs, indicating the advantage of IL-silica composites. The loss modulus was larger than the storage modulus over the entire deformation range for MR electrolyte samples containing silica nanoparticles (0.5, 0.8 and 1.0 wt%), which confirmed a liquid-like behaviour. The values of storage and loss moduli were similar for the MR electrolyte sample with 1.2 wt% silica nanoparticles, illustrating a solid-like behaviour. The dynamic results were consistent with the steady-state shear results, indicating that 1.2 wt% silica nanoparticles engendered a liquid-to-gel transition for the MR electrolyte. Upon application of an external magnetic field, the alignment of the magnetic nanoparticles resulted in solidification, and the storage modulus was significantly larger than the loss modulus. The modulus increased with increasing external field strength (see Fig. S3 in the Supplementary Information).

### Effect of the magnetic field on conductivity

**Figure 3:**
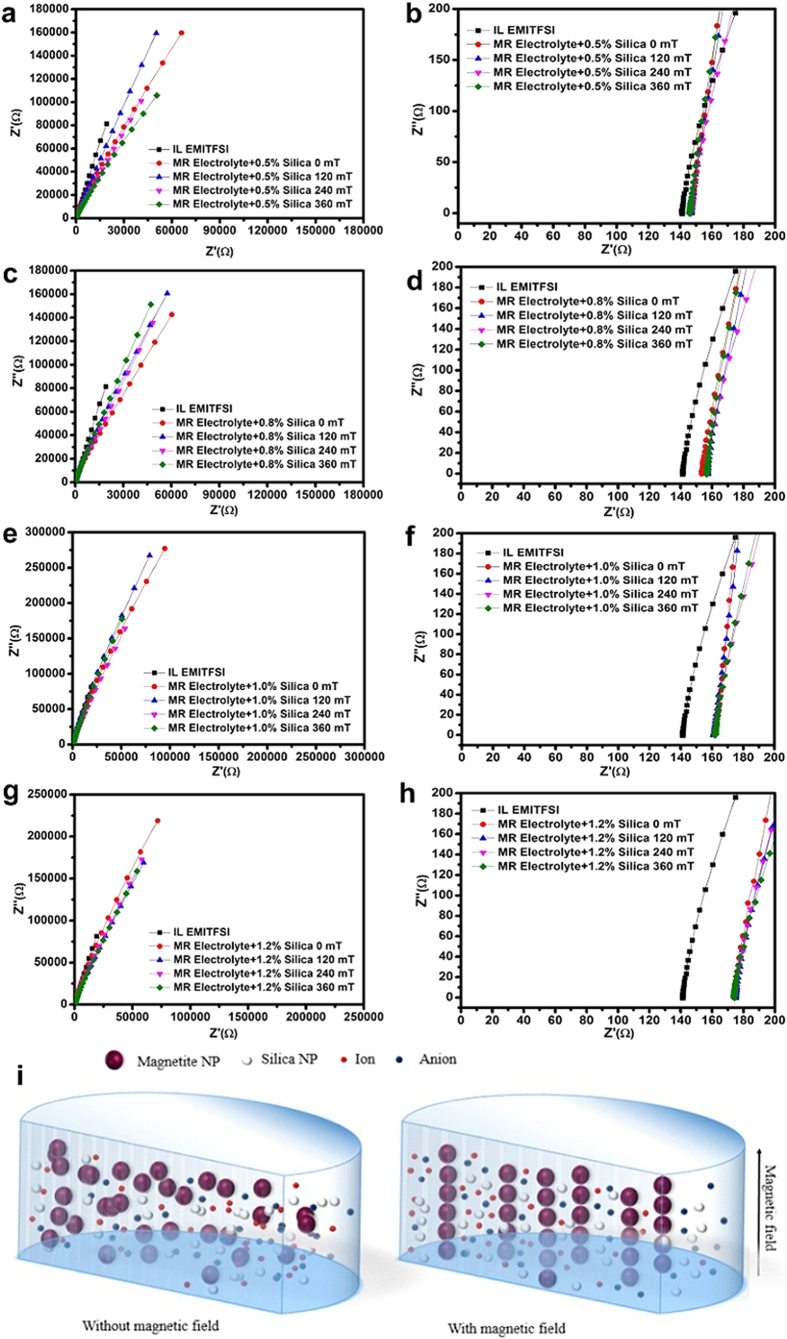
Nyquist plots of MR electrolytes under various external magnetic fields with (**a**) 0.5, (**c**) 0.8, (**e**) 1.0, (g) 1.2 wt% silica nanoparticles and their related expanded view (**b, d, f, h**). (**i**) Proposed schematic of electrical carrier transportation in MR electrolyte under ON/OFF magnetic fields, where the electrical carrier transportation was not substantially affected in either condition.

Figure 3a–h and Table 1 summarise the conductivity values of the MR electrolyte samples. Despite the electrolyte’s increasing viscosity in response to increasing magnetic field strength, the conductivity of the MR electrolyte remained nearly constant (the impedance and conductivity results are also presented in Supplementary Information Fig. S4). The conductivity was only slightly lower than the IL electrolyte. The slight decrease was attributed to the addition of silica nanoparticles, which formed a 3-D network with the IL medium^34^. The results indicated that an external magnetic field could be used to control the mechanical properties by manipulating the phase of the MR electrolyte samples from liquid to semi-solid without any negative effects on conductivity.

### Charge-discharge test

**Figure 4:**
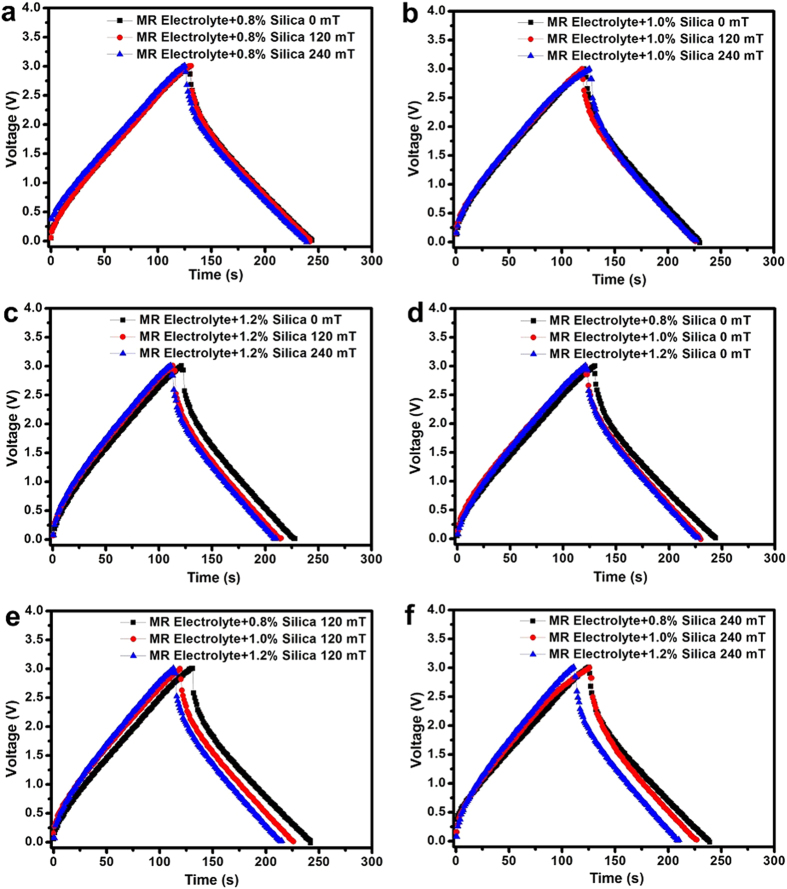
Charge-discharge curves of supercapacitors composed of carbon electrodes using MR electrolytes containing silica-coated magnetic nanoparticles at concentrations of (**a**) 0.8 wt%, (**b**) 1.0 wt%, and (**c**) 1.2 wt% under magnetic strengths of 0, 120 and 240 mT. Charge-discharge behaviour comparison of the supercapacitors using MR electrolytes at the same magnetic fields of (**d**) 0 mT, (**e**) 120 mT, and (**f**) 240 mT.

MR electrolyte samples were used to assemble supercapacitors. Charge/discharge performance comparisons of the supercapacitors using the MR electrolytes with 0.8, 1.0 and 1.2 wt% silica nanoparticles are presented in Fig. 4a–c. No significant decrease in capacity was observed, even when the magnetic field was applied. This charge-discharge experiment should be considered direct evidence that an external magnetic field does not affect the operation of an electronic device that uses an MR electrolyte despite the change in the mechanical properties. The charge-discharge curves of the supercapacitors assembled with various electrolyte compositions under specific magnetic fields are shown in Fig. 4d–f. A larger quantity of silica nanoparticles caused a decrease in overall performance, regardless of the external field strengths.

## Discussion

In this work, a stable, low-viscosity MR electrolyte was fabricated based on a highly conductive IL and silica-coated magnetic nanoparticles. Colloidal stability was demonstrated by sedimentation and redispersion tests. The sample with an addition of 1.0 wt% silica nanoparticles showed good colloidal stability and a maximum change of viscosity when exposed to the magnetic field.

Rheological experiments also indicated that the MR electrolyte samples exhibited a well-defined field-induced phase transition. During the application of an external magnetic field, the conductivity of the MR electrolyte was maintained despite increases in the field-induced viscosity. These results confirmed the novel application of magneto-rheology to electrolyte research. The significance of this unique property of the fabricated MR electrolyte was that the mechanical properties of the electrolyte in electrochemical devices could be controlled from liquid to solid without negatively affecting the conductivity. Thus, devices incorporating this novel electrolyte would be safer.

### Stabilising mechanism of the MR electrolyte

Typically, colloidal particles in carrier media are stabilised through repulsion forces, i.e., electrostatic, steric and/or structural solvation^35^,^36^. If these forces are not comparable to the van der Waals attraction, the particles form aggregates and settle out of the carrier media. In oil-based MRFs, surface surfactant layer substances are often used to introduce electrostatic repulsion and steric repulsion between the dispersed particles^37^. These substances include, but are not limited to, oleic acid, citric acid, tetramethylammonium and polymeric monomers^37^. However, if ILs are used as carrier media, the highly concentrated ions normally suppress the electrostatic effects and steric hindrance becomes a major contribution to the repulsion between particles^38^.

In our work, the sedimentation of a solid phase inside a fluid system depended largely on the difference in density and viscosity of the carrier fluid. Because the EMITFSI used in this study had a constant viscosity of only 50 cP and its density (1.52 g/cm^3^) was considerably lower than that of the dispersed particles (5.1 g/cm^3^), the combined force of steric repulsion or the formation of a solvation structural layer was not sufficient to stabilise the magnetic nanoparticles. Thus, another mechanism was needed to stabilise the silica-coated magnetic nanoparticles in the liquid system^39^, which was the formation of silica-IL composites^40^.

When colloidal particles are added to ILs, interesting phenomena, such as reinforcement, shear thinning, shear thickening, gelation and shear-induced sol-gel transition, can be triggered^41^. Increasing the weight fraction of silica particles in the IL-based system leads to interactions between the silica nanoparticles and ions, thus forming inter-connecting 3-D networks throughout the entire volume of the dispersion^42^. When the amount of silica particles in the IL reach a critical fraction, the silica nanoparticles are no longer dispersed separately but form clusters instead, where a glass transition would occur to form a gelled material^40^. Consequently, this gelation effect, where dispersions evolved from liquid-like behaviour to solid-like behaviour (Fig. 2d), would result in a higher viscosity than that of the original IL. The increased viscosity would be a highly effective cushion to stabilise the silica-coated magnetic nanoparticles in this work.

### Rheological mechanism of the MR electrolyte

Shear thinning is indicative of a disruption in the internal networks after external mechanical loading. This rheological characterisation reflects the evolution of the alignment of the silica-coated magnetic nanoparticles in the MR electrolyte samples when external shear loading was applied. The slight shear-thickening trend at higher shear rates indicated the secondary clustering structure of the inter-connecting silica-IL networks, which were used to stabilise the silica-coated magnetic nanoparticles in the electrolyte system. The liquid system reverted to its original state when the shear loading was removed. When external magnetic fields were applied to the MR electrolyte samples, as described in the Supplementary Information, similar rheological behaviour was observed at higher viscosities. The increasing viscosity is attributed to the orderly alignment of dispersed silica-coated magnetic nanoparticles in the presence of a magnetic field.

### Proposed mechanism of conductivity of the MR electrolyte

Ionic conductivity is determined by ionic mobility and carrier concentration^43^. In our conductivity tests, there were two components that could affect the transportation of ions (compared with the neat IL): the silica-IL interface and the alignment of silica-coated magnetic nanoparticles under an external magnetic field. The interconnecting silica-IL cluster would consume some ions, an equilibration occurring between the formation and destruction of the composite structure, which was why the MR electrolyte samples were less conductive when higher fractions of silica nanoparticles were added. In our study, the gelation effect introduced to maintain the stability of the electrolyte dispersion was kept at a minimum, so the sample remained liquid rather than transforming into a gel. Thus, the reduction of effective ionic carriers was minimised^26^. When no external magnetic field was applied to the MR electrolyte, the magnetic nanoparticles dispersed randomly due to the combined effects of Brownian forces and the stabilising network interface between the silica particles and IL, and thus, the conductive elements that carry the charges flowed smoothly throughout the liquid system.

When an external magnetic field was applied to the MR electrolyte in a direction perpendicular to the testing cell, the field-induced magneto-static force would result in a bipolar effect that binds the sensitive magnetic nanoparticles into elongated alignment^44^. This bipolar effect is achieved when magnetic nanoparticles are placed in a uniform magnetic field, creating magnetic moments due to the magnetisation of all particles. These magnetic moments lead to interactions between adjacent particles on both sides of the particle, resulting in finite alignment lengths. The transition leads to significant changes in rheology and phase of the MR electrolyte, as shown in Fig. 2. There could be a number of possible structures of the field-induced aggregates, including spheroidal, cylindrical, and layered particle aggregates or free single chain, columnar and other labyrinthine structures^44^,^45^,^46^. The particular structure formed depends on the initial volume fraction and the parameter λ, i.e., the ratio of the magnetic field energy to the thermal force^44^. In this study, considering the dilute concentration of magnetically sensitive nanoparticles used and the effective steric repulsion force generated by the silica-IL composite^38^, the magnetically sensitive silica-coated magnetic nanoparticles would form a stable chain-like or column-like structure along the direction of an applied magnetic field. The orderly geometric structure in the dispersion system acted as a sponge, where the remainder of the space is filled with IL. Because the unfilled space is considerably larger than the ions, their mobility is not affected by the alignment of the magnetic nanoparticles, which explains why the conductivity was not significantly affected upon the application of magnetic fields. A schematic of the mechanism is presented in Fig. 3i. This result explains why conductivity was not significantly changed and sheds light on the optimisation of the microstructure of novel solid electrolytes based on magneto-rheology by introducing a permanent matrix. Because the transportation of effective charge carriers was not affected by external magnetic fields applied to MR electrolytes, the effect of temperature on an MR electrolyte would be the same as for IL, where higher temperatures would accelerate the charge carrier motion, leading to increased conductivity^47^.

## Methods

### Synthesis of the core/shell particles

Silica-coated iron (II.III) oxide nanoparticles (Fe_3_O_4_) were used to generate the electrolyte samples. They were prepared through the hydrolysis of tetraethyl orthosilicate (TEOS) in an alkaline environment. The chemicals used in this synthesis include magnetic nanoparticles with a unit size of 50–100 nm (Alfa Aesar, United Kingdom), TEOS, (98%, Sigma Aldrich, US), aqueous ammonia (25 wt%, Sigma Aldrich, US) and ethanol (100%, Sigma Aldrich, US). All reagents were used without follow-up processing or purification.

First, 1 g of Fe_3_O_4_ nanoparticles was dispersed in 16 mL of deionised water and 80 mL of ethanol in an ultrasonic water bath. Then, 12 mL of aqueous ammonia (25 wt%) was added to the dispersion, and the mixture was stirred vigorously at room temperature. Next, 1.8 mL of TEOS was added dropwise basis over a 24 h period while the solution was stirred continually to ensure the Fe_3_O_4_ nanoparticles were completely coated. After this hydrolysis reaction was completed, the core shells Fe_3_O_4_@SiO_2_ nanoparticles could be obtained with a magnet. The particles were washed several times in deionised water and then dried at 50°C.

### Observation of sedimentation

To characterise the sedimentation, the suspensions were stored in test tubes for one week, and the heights of the sediment-supernatant interfaces were monitored and recorded. A higher solid phase indicated a more stable electrolyte.

**The protocol for the redispersion experiment** was as follows: A 50 s^−1^ pre-shear was applied to the liquid sample for 2 min, followed by a standby time of 1 min. A constant shear stress of 20 Pa was then applied to the liquid sample, and the corresponding shear rate was recorded. Another 3 min pre-shear was then applied to ensure the particles’ complete redispersion. These steps were then repeated after 15 min, 30 min, 60 min and 24 h. The derived shear rates were recorded in a log ramp manner, with increasing intervals from 0.1 s to 10 s recorded logarithmically. The resultant shear rate values were then compared to determine the state of aggregation^21^. The redispersion experiment and all other rheological experiments were carried out on an advanced stress controlled rheometer (Anton Paar MCR 301, Germany). The unified geometry applied in these rheological tests was a pair of parallel plates with a 0.3 mm gap. All experiments were carried out at 20 °C.

**The protocol for the magnetic sweep experiments** was as follows: A 50 s^−1^ pre-shear was applied for 1 min, followed by a 1 min standby time. The linear ramp of a perpendicular external magnetic field ranging from 0 to 660 mT was then applied with a constant shearing of 0.01 s^−1^, and the instantaneous viscosity was recorded.

**The shear rate sweep experiments** began with a 50 s^−1^ pre-shear for 1 min. During the standby minute where no shear loadings were applied, the specific magnetic strength was applied in the perpendicular direction to trigger the magneto-rheological effect. A log ramp shear rate from 0.01 to 500 s^−1^ was then applied to the fluid, and the shear stress and viscosity were recorded.

**Oscillatory dynamic tests** began with a 50 s^−1^ pre-shear for 1 min. Oscillatory shear loading was applied to the MR electrolyte samples with increasing peak strain from 0.01–1,000% with a specific external magnetic field in the perpendicular direction, and the angular frequency was a constant 5 rad/s.

**Impedance tests** between two quadrilateral stainless steel electrodes were carried out to determine the conductivity of the MR electrolyte samples. A pair of permanent magnets was used to supply a magnetic field. A gauss meter was used to record the field strength at each test while the field strength was changed by adjusting the distance between the pair of magnets. A series of impedance tests were carried out on the electrolyte samples with 0.5, 0.8, 1.0 and 1.2 wt% silica nanoparticles. The conductivities under various field strengths can be calculated as shown: *α* = *L/AR*_c_, where *L* is the gap between the electrodes, *A* is the conductive area, and *R*_*c*_ is the resistance value at the highest frequency in the impedance tests.

**Charge/discharge performance** were all conducted at a current density of 1 A/g. The charge-discharge curves of the supercapacitors composed of a pair of carbon electrodes and different MR electrolytes were collected under different external magnetic fields.

## Additional Information

**How to cite this article**: Ding, J. *et al.* Novel reversible and switchable electrolytes based on magneto-rheology. *Sci. Rep.* **5**, 15663; doi: 10.1038/srep15663 (2015).

## Supplementary Material

Supplementary Information

Supplementary Information

Supplementary Information

## Figures and Tables

**Table 1:** Summary of impedance test.

	**0 mT**		**120 mT**		**240 mT**		**360 mT**	
	**Rc (Ω)**	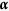 **(mS/cm)**	**Rc (Ω)**	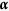 **(mS/cm)**	**Rc (Ω)**	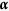 **(mS/cm)**	**Rc (Ω)**	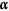 **(mS/cm)**
IL	141.3	5.83	141.4	5.83	141.1	5.83	141.3	5.83
MR electrolyte + 0.5% silica	146.4	5.69	146.9	5.67	146.6	5.68	146.0	5.71
MR electrolyte + 0.**8**% silica	153.5	5.44	157.2	5.32	156.0	5.38	156.7	5.35
MR electrolyte + 1.0% silica	162.0	5.13	160.8	5.17	161.8	5.14	161.9	5.14
MR electrolyte + 1.2% silica	174.7	4.72	174.9	4.71	174.3	4.74	173.9	4.76
