# Supplementary material for: Novel reversible and switchable electrolytes based on magneto-rheology
Source: Sci Rep. 2015 Oct 23;5:15663. doi: 10.1038/srep15663 (PMC4616165; doi:10.1038/srep15663)
Supplement: Supplementary Information [file srep15663-s1.pdf]

Title: Novel reversible and switchable electrolytes based on magneto-rheology

Authors: Jie Ding[1]*, Gangrou Peng[2], Kewei Shu[3], Caiyun Wang[3], Tongfei Tian[2], Wenrong Yang[4], Yuanchao Zhang[4], Gordon G Wallace[3], and Weihua Li[2]*

[1] Land Division, Defence Science and Technology Organisation, 506 Lorimer Street, Fishermans Bend, VIC 3207, Australia, [2] School of Mechanical, Material and Mechatronic Engineering, University of Wollongong, Wollongong, NSW 2522, Australia, [3] ARC Centre of Excellence for Electromaterials Science, Intelligent Polymer Research Institute, University of Wollongong, Wollongong, NSW 2522, Australia, [4]School of Life and Environmental Sciences, Deakin University, Geelong, VIC 3217, Australia.

*Correspondence and requests for materials should be addressed to J. D. (Jie.Ding@dsto.defence.gov.au), and W. H. L. (weihuali@uow.edu.au)

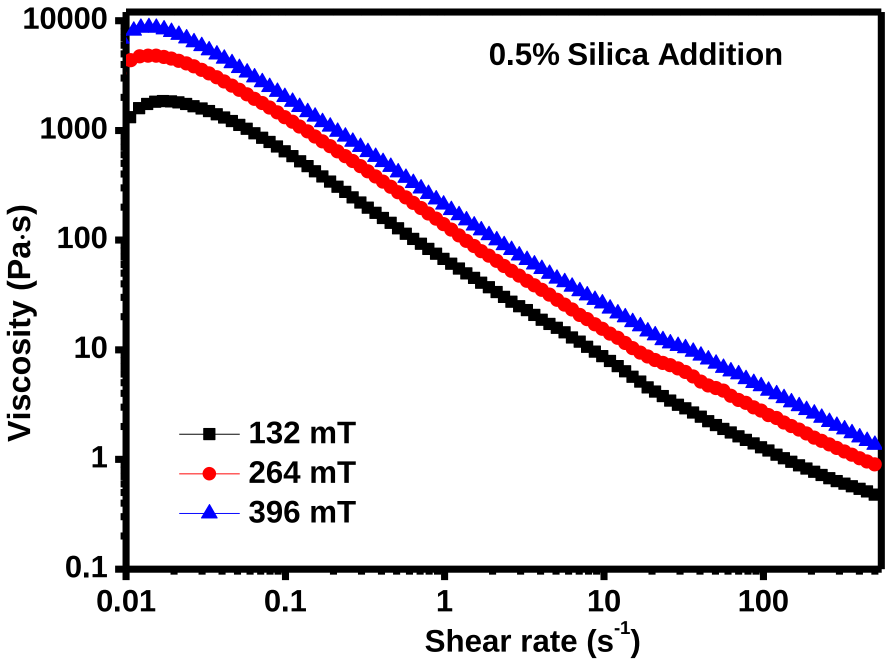

Figure S1. (a) Shear rate sweep experiment for the MR electrolyte sample containing 0.5 wt% silica nanoparticles under field strengths of 132, 264 and 396 mT.

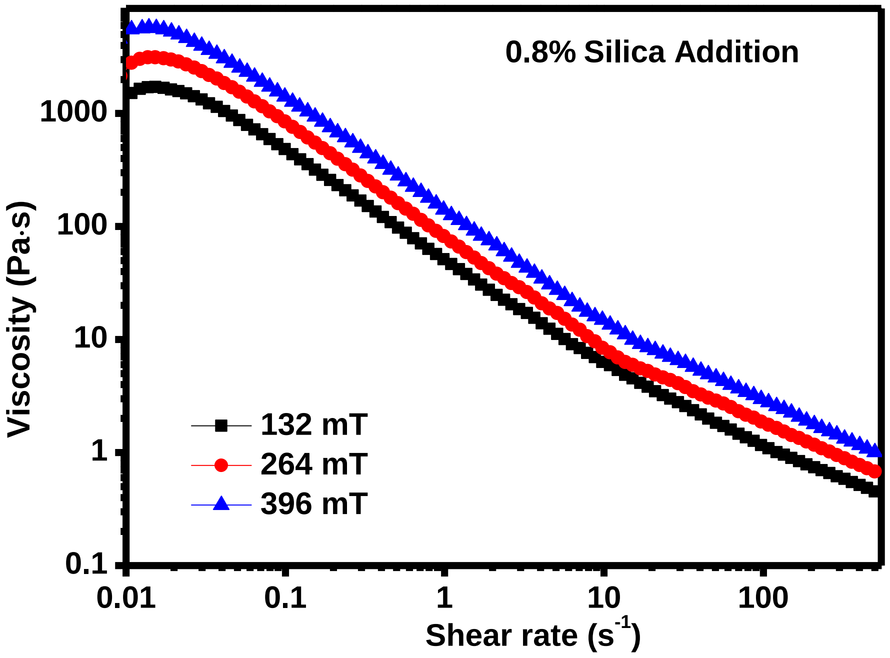

Figure S1. (b) Shear rate sweep experiment for the MR electrolyte sample containing 0.8 wt% silica nanoparticles under field strengths of 132, 264 and 396 mT

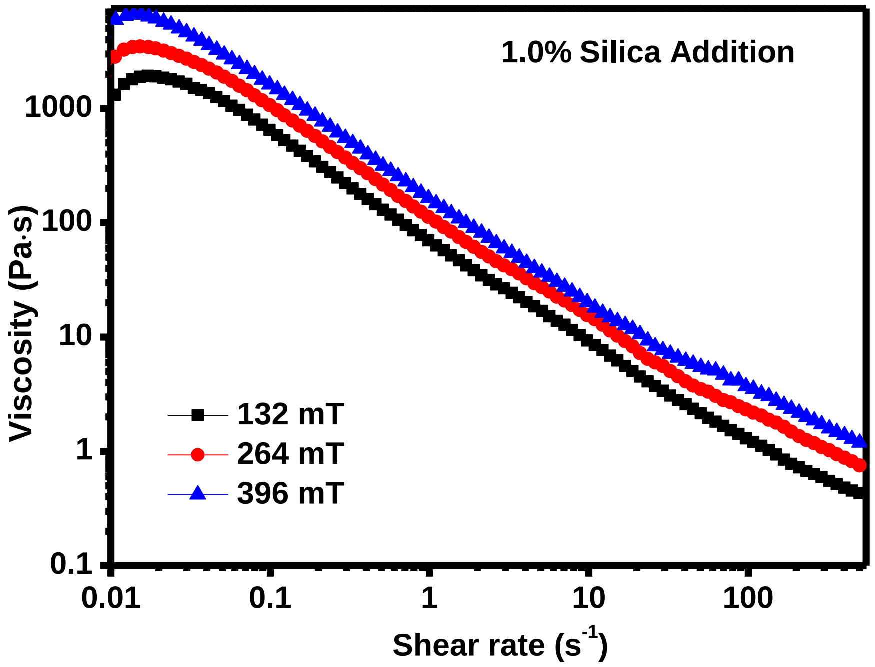

Figure S1. (c) Shear rate sweep experiment for the MR electrolyte sample containing 1.0 wt% silica nanoparticles under field strengths of 132, 264 and 396 mT.

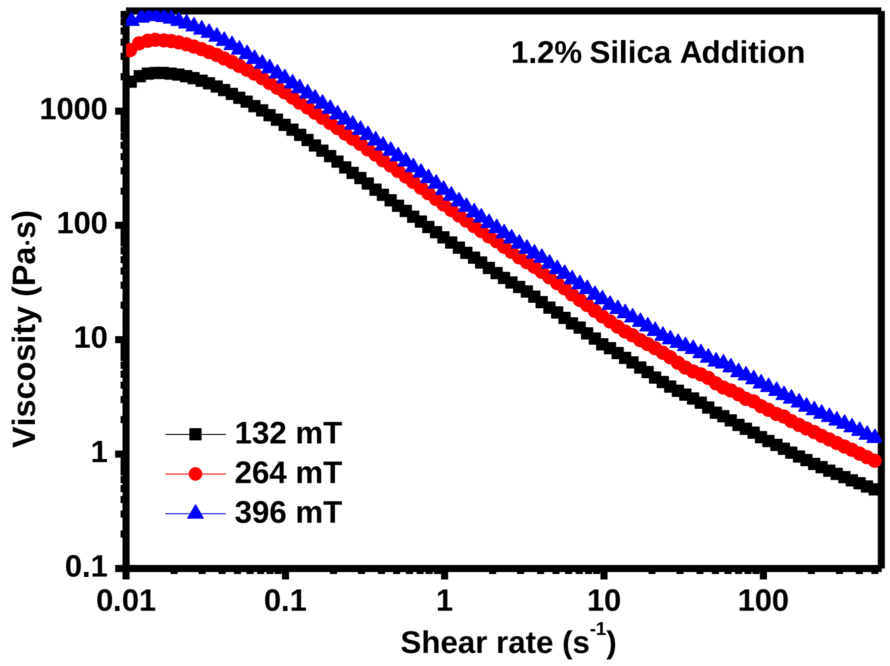

Figure S1. (d) Shear rate sweep experiment for the MR electrolyte sample containing 1.2 wt% silica nanoparticles under field strengths of 132, 264 and 396 mT.

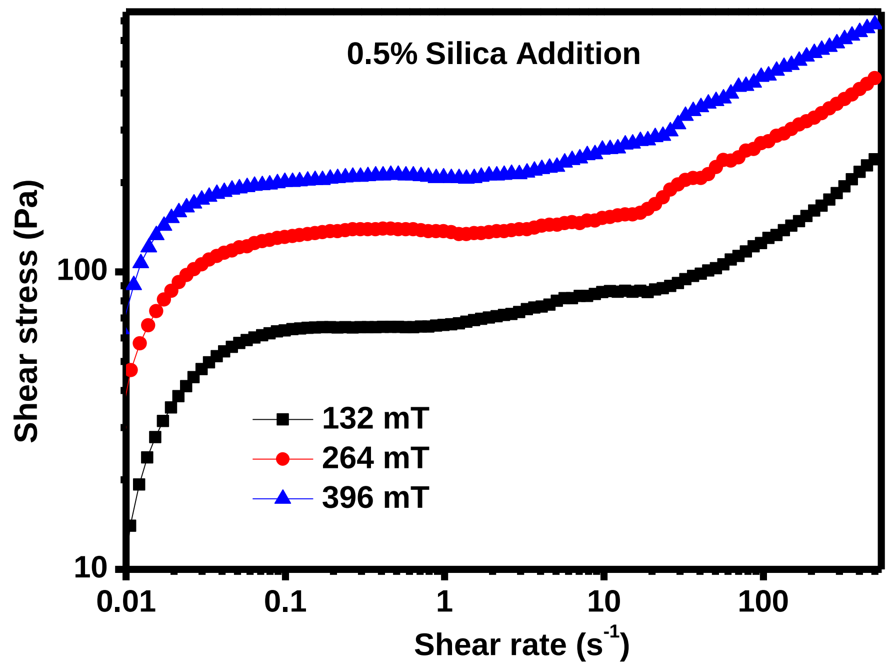

Figure S2. (a) Shear stress vs. Shear rate flow curve for the MR electrolyte sample containing 0.5 wt% silica nanoparticles under field strengths of 132, 264 and 396 mT.

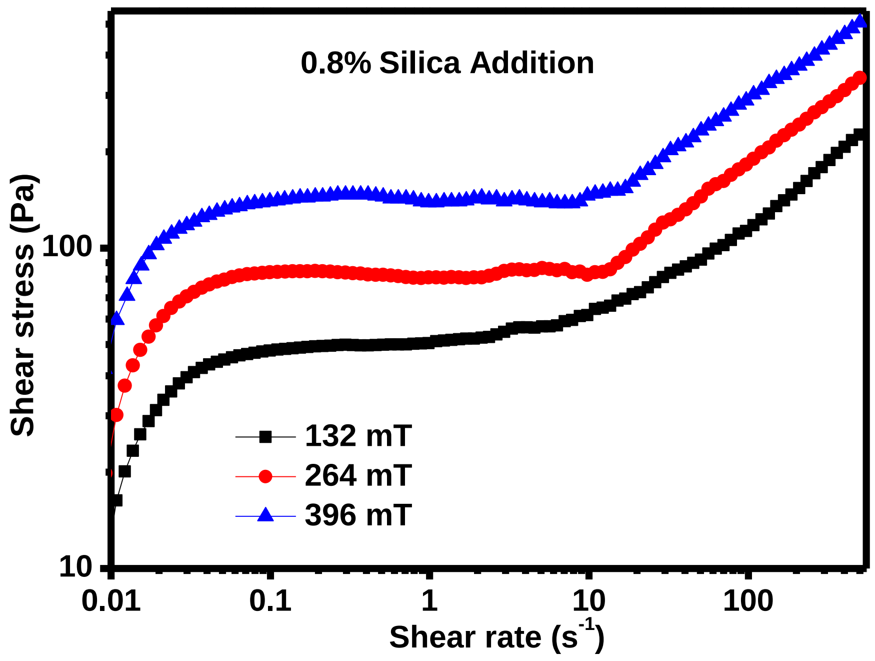

Figure S2. (b) Shear stress vs. Shear rate flow curve for the MR electrolyte sample containing 0.8 wt% silica nanoparticles under field strengths of 132, 264 and 396 mT.

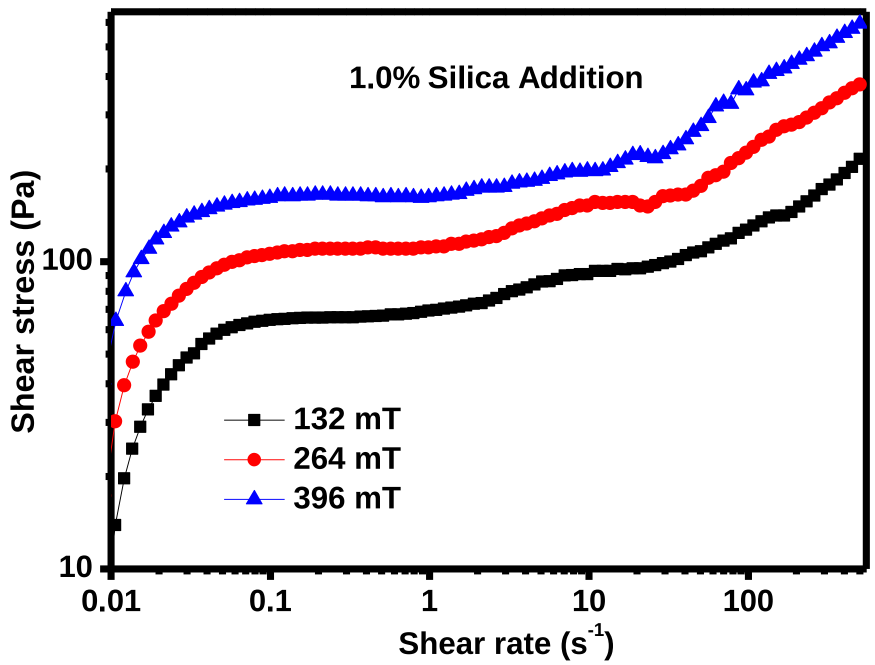

Figure S2. (c) Shear stress vs. Shear rate flow curve for the MR electrolyte sample containing 1.0 wt% silica nanoparticles under field strengths of 132, 264 and 396 mT.

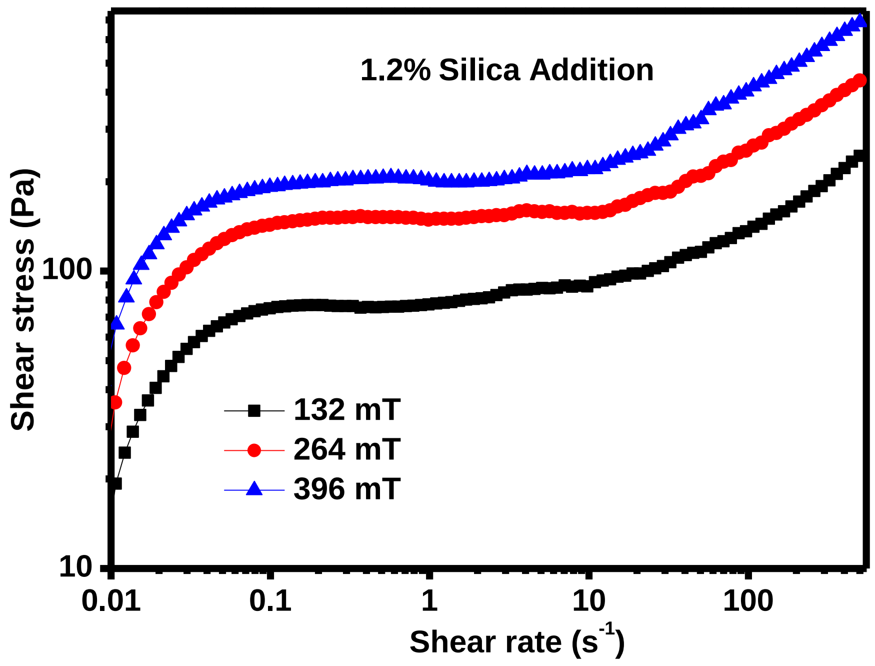

Figure S2. (d) Shear stress vs. Shear rate flow curve for the MR electrolyte sample containing 1.2 wt% silica nanoparticles under field strengths of 132, 264 and 396 mT.

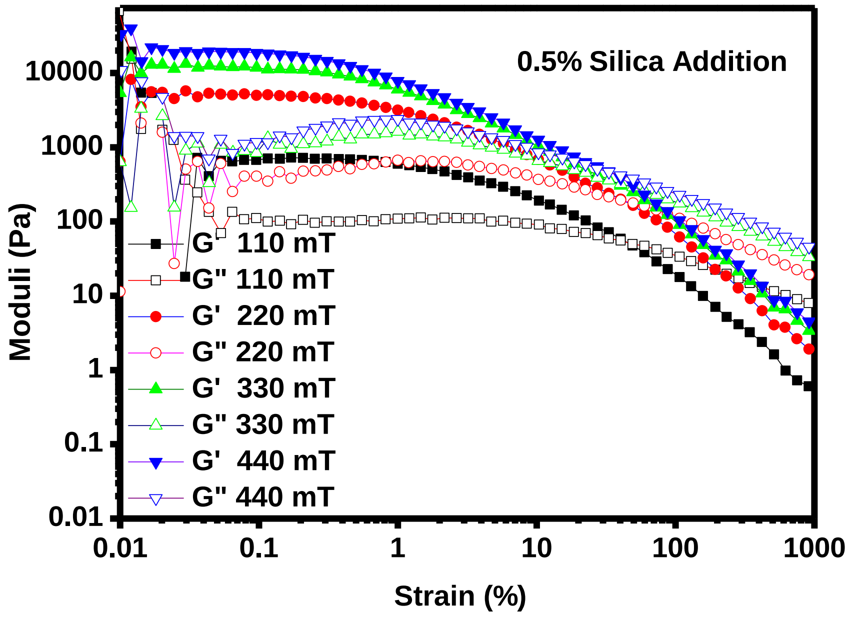

Figure S3. (a) Shear strain sweep of oscillatory dynamic experiment for the MR electrolyte containing 0.5 wt% silica nanoparticles under field strengths of 110, 220 330 and 440 mT.

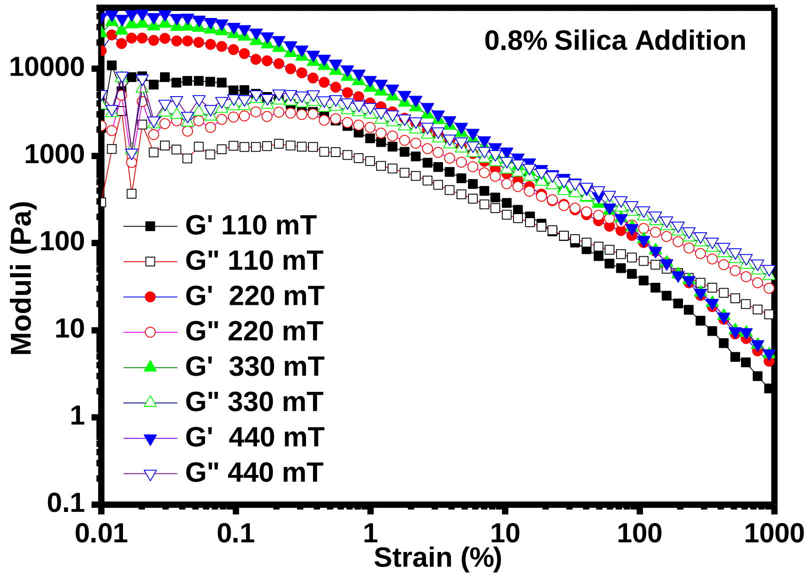

Figure S3. (b) Shear strain sweep of oscillatory dynamic experiment for the MR electrolyte containing 0.8 wt% silica nanoparticles under field strengths of 110, 220 330 and 440 mT.

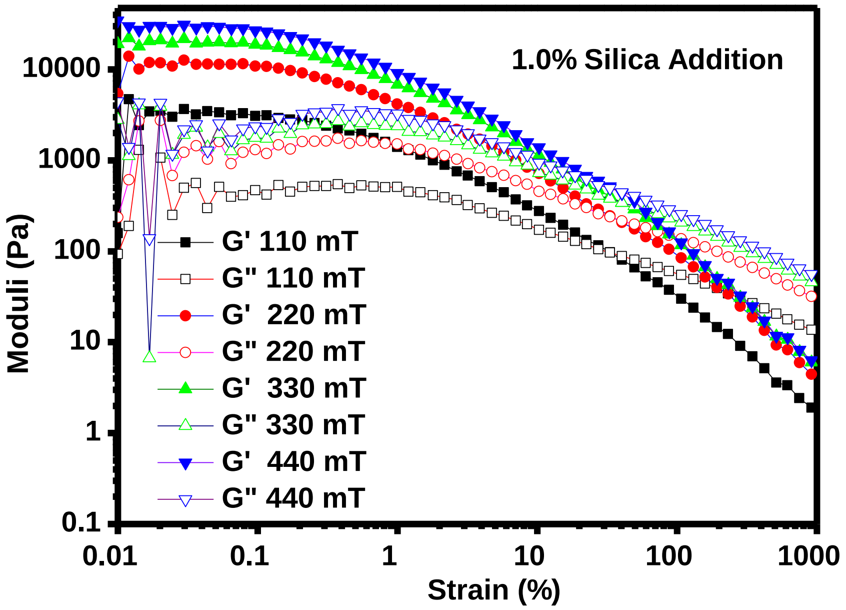

Figure S3. (c) Shear strain sweep of oscillatory dynamic experiment for the MR electrolyte containing 1.0 wt% silica nanoparticles under field strengths of 110, 220 330 and 440 mT.

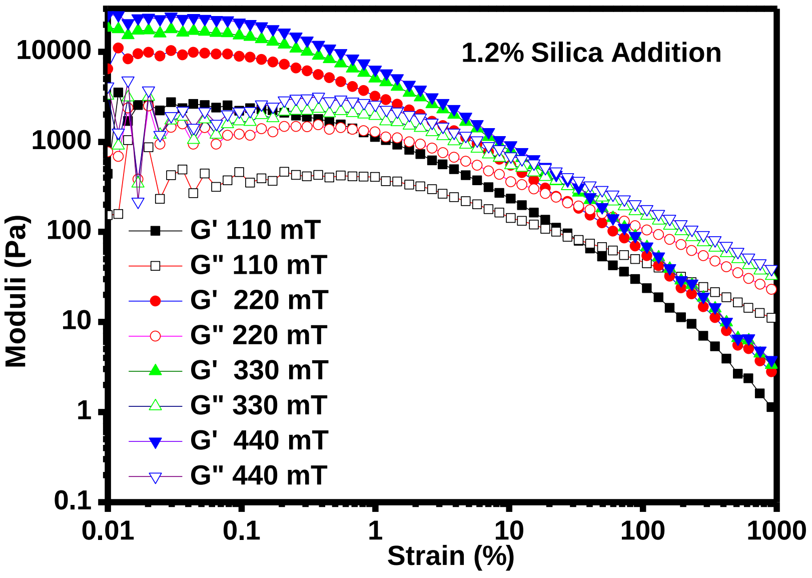

Figure S3. (d) Shear strain sweep of oscillatory dynamic experiment for the MR electrolyte containing 1.2 wt% silica nanoparticles under field strengths of 110, 220 330 and 440 mT.

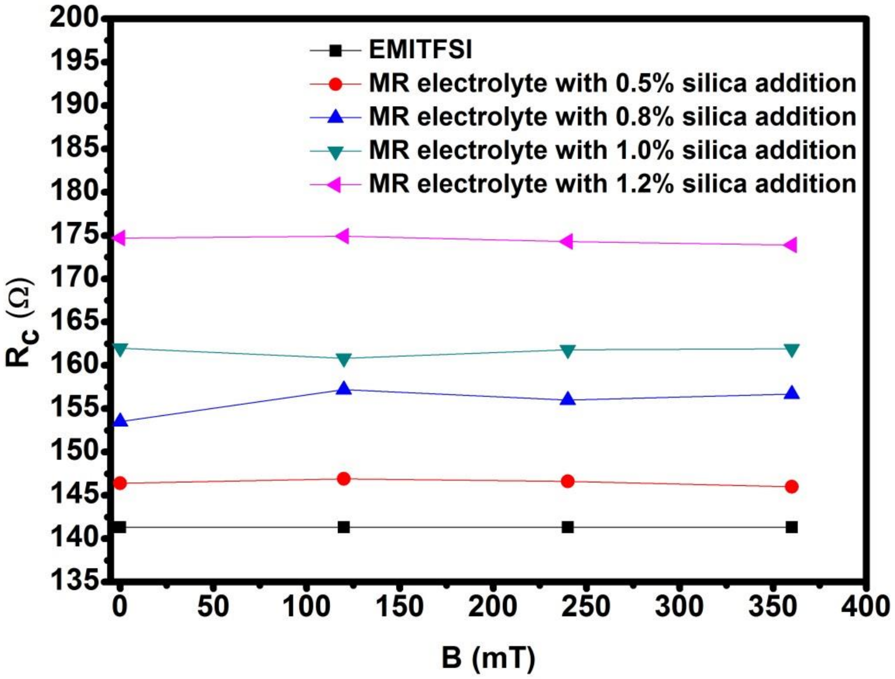

Figure S4. (a) Bulk resistance (estimated from EIS) of pure EMITFSI and MR electrolyte samples containing 0.5, 0.8, 1.0 and 1.2 wt% silica nanoparticles under field strengths of 0, 120, 240 and 360 mT.

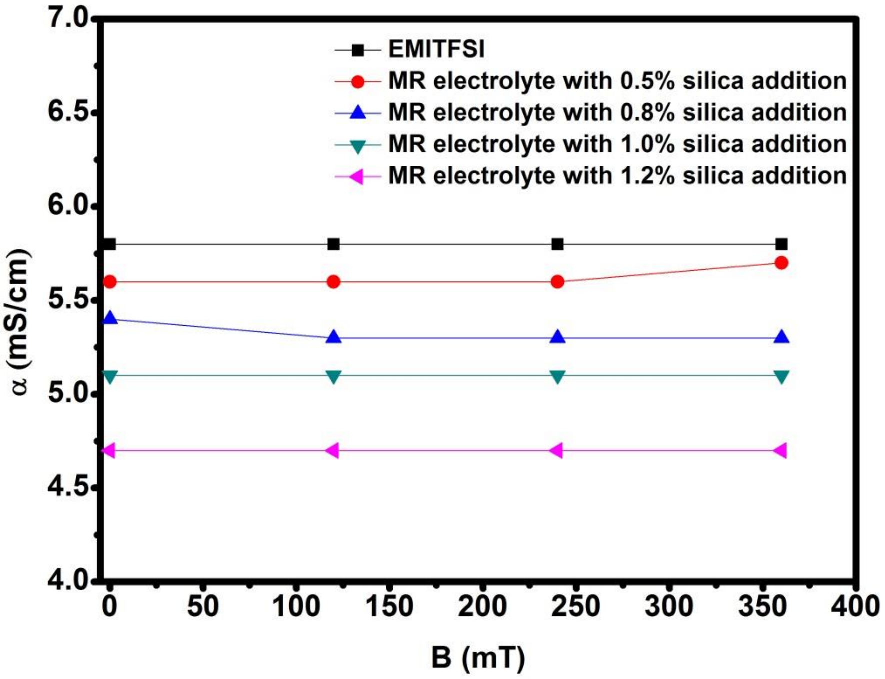

Figure S4. (b) Conductivity of pure EMITFSI and MR electrolyte samples containing 0.5, 0.8, 1.0 and 1.2 wt% silica nanoparticles under field strengths of 0, 120, 240 and 360 mT.

Two video clips are also presented to demonstrate the effect of the external magnetic field on the silica-coated magnetite nanoparticles and the prepared MR electrolyte samples.

Video S1. Magnetic field to particle shows that the silica-coated magnetic nanoparticles could still be collected using a magnet stick. This behaviour means that the treated magnetite nanoparticles were still sensitive to external magnetic fields.

Video S2. Magnetic field to MR electrolyte shows that the MR electrolyte sample solidifies under external magnetic fields and resumes a liquid state when the external magnetic field is removed.
